# Interactions among Ecological Factors That Explain the Psychosocial Quality of Life of Children with Complex Needs

**DOI:** 10.1155/2010/404687

**Published:** 2010-06-07

**Authors:** Sandy Thurston, Louise Paul, Chenglin Ye, Patricia Loney, Gina Browne, Lehana Thabane, Peter Rosenbaum

**Affiliations:** ^1^Children's Treatment Network, Simcoe/York, ON, Canada L4M 2Y1; ^2^System-Linked Research Unit on Health and Social Service Utilization, Faculty of Health Sciences, McMaster University, Hamilton, ON, Canada L8P 0A1; ^3^Children's Health Research Institute, McMaster University, Hamilton, ON, Canada L8N 3Z5; ^4^CanChild, McMaster University, Hamilton, ON, Canada L8S 1C7

## Abstract

*Purpose*. To explore the associations and interactions among ecological factors and explain the psychosocial quality of life of children with complex needs. *Methods*. In this cross-sectional survey consenting parents were identified by the Children's Treatment Network. Families were eligible if the child from 0 to 19 years, resided in Simcoe/York, and there were multiple family needs. Regression analysis was used to explore associations and interactions. *n* = 429. *Results*. Younger children, without conduct disorder, without hostile and punitive parenting and with low adverse family impact demonstrated the highest levels of psychosocial quality of life. Statistically significant interactions between processes of care and parent variables highlight the complexity of real life situations. *Conclusions*. It is not possible to fully understand the child's psychosocial quality of life in complex needs families by considering only simple associations between ecological factors. A multitude of factors and interactions between these factors are simultaneously present and the care of these families requires a holistic approach.

## 1. Introduction

Advances in biomedical science and technology have brought the hope of survival to more and more children and youth with complex needs [1]. Many of them now live into adulthood and function in the community [2]. Accordingly; a growing interest has emerged in the children's quality of psychosocial life in spite of the continuing or deteriorating limits to their physical and/or cognitive function [3]. The psychosocial quality of life is the individual's report of their age appropriate social and emotional participation in life in the context of the culture and value systems in which they live [4]. Treatment efforts have traditionally focused on the physical deficits of the child. The psychosocial well-being of children with complex needs, however, appears to be more strongly linked with child behaviour, parent and family impact variables [5]. Studies have shown that the psychological well-being of special needs children is associated with lower pain levels, lower parent distress, fewer symptoms of ADHD, no comorbid psychiatric diagnoses, fewer behavioral difficulties, less receipt of rehabilitation and more accepting/autonomous parenting styles [6–9]. 

Current thinking has moved from studies of simple univariate associations between variables to hypotheses that acknowledge the interplay among ecological and transactional multilevel factors within the child, parent, family, school, and health care provider(s). It is hypothesized that there are types of causalities (potentialities and instigators) and levels of multiple other associated factors within the child/parent/family/social and health service environments that interact to explain the quality of life in a child with complex needs [10].

Using the evolving Bronfenbrenner and Morris [10] bio-ecological model of human development, the principal components and the dynamic interactive relationships among them can be described in relation to the child's psychosocial health. The particular forms of interaction between the children and their environments that operate over time (“proximal processes”) are posited as the primary mechanisms producing human development. The power of such processes to influence development has been shown to vary substantially as a function of the characteristics of the developing, immediate and more remote environmental contexts, and the time periods in which the proximal processes take place. As stated by Bronfenbrenner [11] “… in ecological research the principal main effects are likely to be interactions.” Bronfenbrenner's [10] general hypothesis is that the developmental impact of proximal processes growing up in disadvantaged or disorganized environments is greater for outcomes reflecting developmental dysfunction. By contrast, for outcomes indicating developmental competence, proximal processes are posited as having likely greater impact in more advantaged and stable environments. 

**Figure 1:**
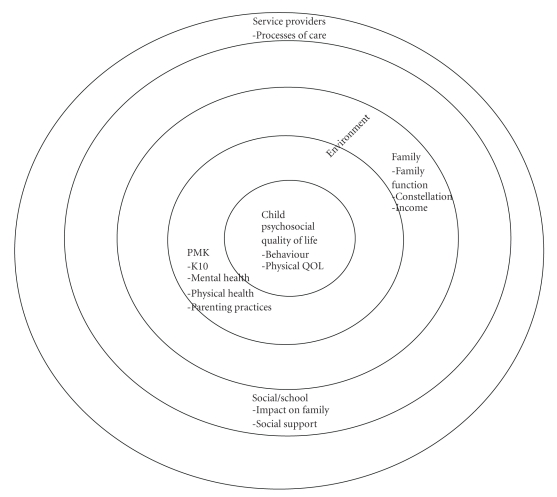
Bioecological model of key factors and their interactions with child psychosocial function.

Specifically, children with complex needs experience a constellation of physical limitations and behaviours over time (age) and interact within their environment (parent, family, school, social and health providers) (Figure 1). Concurrently, parents may have their own social/emotional/physical issues yet they must respond to and live with the child's behaviour in addition to physical limitations as the child develops (this is the essence of parenting). Family, school and treatment providers also respond to the child and parent involving them in a complex interplay of environmental factors. We hypothesize that the reciprocal influence between the child's behavior, their immediate parenting/family environments, parent report of impact on the family, parent depression/anxiety and well-being, and family function influences the child's psychosocial function. Supportive and comprehensive processes of care from health care providers may help to influence child psychosocial function when parents exhibit poor physical or mental health, and family outcomes. A better understanding of the interplay among these variables would help highlight the emphasis to be taken in child and family rehabilitation if the objective is to maximize the child's quality of life (physical and psychosocial) and promote the family's ability to live with their child's disability [12]. Further, an understanding of the service provider processes of care that influence the child's psychosocial quality of life when the child, parent or family have additional psychosocial circumstances and behaviours would offer specific guidance to providers. As stated so clearly by Koroloff and Friesen [13] “family strength and family focused models and perspectives embraced by current service delivery demand new research approaches.” 

The purpose of this paper is to test this conceptual framework of ecological factors within the child, parent, family, social and health services environment that could potentially interact to explain the quality of psychosocial life in a heterogeneous sample of children with complex needs.

## 2. Methods

### 2.1. Research Setting

This descriptive study is part of a cohort study examining the effects and expenses of more and less integration of services that provide treatment and rehabilitation for children with complex needs. The cohort is enrolled in the newly modeled Children's Treatment Network (CTN) of Simcoe/York counties in Ontario. The CTN approach is unique in that it is based on the collaboration of numerous existing autonomous local service agencies utilizing the service co-ordination and electronic record functions of the CTN. Ethics approval was obtained for the study by the Research Ethics Board of McMaster University.

#### 2.1.1. Study Design and Procedures

This was a cross-sectional survey of families with a special needs child enrolled in the CTN from May to December 2007. Families were deemed eligible if the child's age was between 0 and 19 years, were residents of Simcoe/York, and there were multiple needs within the family (child's special needs and/or families needs, for example, a parent's medical or mental health problem). The consenting parent/guardian most knowledgeable (PMK) returned a signed consent form to McMaster University indicating their willingness to participate. The PMK then completed a telephone interview (1 hour) by one of three trained interviewers from McMaster University. The size of this convenience sample of PMK completing the interview was 445. 

### 2.2. Measures 

#### 2.2.1. Child Quality of Life

The PedsQL is a generic measurement system developed by Varni et al. [14]. The shortened version consists of 15 items comprising three core scales and addresses the physical (5 items), emotional (4 items), social (3 items) and school functioning (3 items) [15]. Parent proxy report formats were used for children aging from 2 to 18 due to the inclusion of children with limited cognitive or communicative abilities. Each item for ages from 8 to 18 asks how much of a problem it has been during the past month on a five point scale (0-“never a problem” to 4-“almost always a problem”). For children aged 5 to 7, the scale is modified to 0-“not a problem”, 2-“sometimes a problem” and 4-“a lot of a problem.” Items are reverse scored and linearly transformed to a 0–100 scale so that higher scores indicate better quality of life. Psychosocial Quality of Life (PsychQL) is computed as the sum of the Emotional, Social and School scale scores (10 items, range 0–100). Reliability and validity of the shortened version have been documented [15].

#### 2.2.2. Child Behaviour

Behaviour was measured using the NLSCY Behaviour questionnaire for children aged from 0 to 19 [16]. The questionnaire asks about how the child seems to feel or act regarding age-specific behaviours such as getting into fights, inability to sit still, and worrying. The parent is asked to rate the specific behaviour from 1-“never” to 3-“often.” Behaviour subscales include hyperactivity/inattentive, prosocial, anxiety/emotional disorder, conduct disorder/ physical aggression, indirect aggression and property offence. Items differ for age groups 0-1 years, 2–5 years and 6–19 years. Specifically, questions pertaining to aggression, property offense and prosocial behaviour do not apply to the younger age groups. Internal consistency is reported by subscale and age group (Cronbachs alpha 0.68–0.84) [17].

#### 2.2.3. Health of PMK

The Kessler scale (K10) [18] measures PMK symptoms of depression and anxiety, a frequent accompaniment of depression. Ten questions measure the frequency of feeling: sad, nervous, restless, hopeless, worthless, everything was an effort, tired for no good reason, so nervous that nothing could calm down, fidgety, so restless, could not sit still, or depressed during the past month. Chronic aspects of distress in the past month are examined on a five point scale (1-“all of the time” to 5-“none of the time”). Reliability and validity have been documented [19]. Scores range from 10–50 where scores ≤19 indicate no clinically important level of distress, 20–24 indicates mild distress, 25–29 moderate distress and 30–50 severe distress. 

#### 2.2.4. Parent Wellbeing

Parents were asked to rate their mental, physical health and general life satisfaction on a five point scale (1-“very satisfied” or “excellent” to 5-“very dissatisfied” or “poor”). These questions were taken from the Canadian Community Health Survey (CCHS 2.2) [20].

#### 2.2.5. Caregiver Burden

The Impact on Family (IOF) Scale determines the effects of a chronic illness on parents and families. Parents respond on a four-point scale, to the degree that statements apply to their family (1-“strongly agree” to 4-“strongly disagree”) [21]. The revised IOF scale (15 items) has been validated [22, 23]. Statements cover four dimensions: financial burden, family/social impact, personal strain, and mastery (e.g., fatigue is a problem, see family and friends less, need to change plans at last minute, little desire to go out). 

#### 2.2.6. Parenting Practices

The NLSCY Parenting Scale was used and it consists of twenty-five questions adapted from the validated Parenting Practices Scale [24]. The following four parenting behaviours were measured: positive interaction (praise, play), hostility (anger, discipline), consistency (follow through) and punitive (yelling, physical punishment). PMK rates each item (e.g., “Do something special with your child that he/she enjoys”) in terms of frequency from 0-“never” to 4-“many times each day.” Higher scores indicate greater frequencies for each type of parenting behaviour. Internal consistency is reported by subscale and age group (Cronbachs alpha 0.39–0.75) [17].

#### 2.2.7. Social Support

The level of the social support of the PMK was assessed using an eight-item shortened version of the Social Provisions Scale [25]. Different social support constructs were measured; guidance, reliable alliance (i.e., feeling assured that others would be available to offer practical help), and attachment. PMK rates each item along a four point scale from 0-“strongly disagree” to 3-“strongly agree.” Higher scores represent greater social support. The reliability and validity of the measure have been reported [25].

#### 2.2.8. Family Functioning

Thirteen items from the NLSCY population survey [16], based on a subscale of the McMaster Family Assessment Device [26] were used to gather information on various aspects of family functioning (problem solving, communication, roles, affective responsiveness, affective involvement, behaviour control). PMK rated each item (e.g., “We avoid discussing our fears or concerns”) along a four point scale from 1-“strongly agree” to 4-“strongly disagree.” Negatively oriented items are reverse scored so that higher scores represent greater family dysfunction. The measure has internal consistency (Chronbach's alpha 0.86) [26]. Scores range from 0–36 with scores ≥15 indicates family dysfunction.

#### 2.2.9. Parents' Perception of Family-Centeredness of Services

The Measure of Processes of Care (MPOC-20) is a 20-item, well-validated and reliable self-report measure of parents' perceptions of the extent to which the services they and their child receive is family centered [27, 28]. Respondents use a seven-point scale to describe the extent to which they experience service provider behaviours across five domains with response options ranging from 1-“never” to 7-“to a great extent.” The five domains are: Enabling and Partnerships, Providing General Information, Providing Specific Information, Comprehensive and Coordinated Care and Respectful and Supportive Care. A “not applicable” category is included. MPOC scales have good internal consistency (Cronbach alpha 0.77–0.96) [29]. 

#### 2.2.10. Demographics of the Child

Includes child's age, gender, grade and PMK report of the main medical and other important diagnoses. 

#### 2.2.11. Demographics of the Family

A standard form including spiritual or faith orientation, ethnicity and languages was selected from the Canadian National Longitudinal Study on Children and Youth (NLSCY) that also includes community dwelling disabled children [16]. Sociodemographic data were gathered on the PMK gender, age and educational level as well as on household income and family status. 

## 3. Statistical Analysis

 Descriptive statistics (numbers, percentages, means and standard deviations) were calculated for all child and family variables. The child and PMK variable had a changing number of participants for several reasons. The behaviour subscale measures have different numbers of items applicable to different age groups. The prosocial behaviour scale items for children and youth from 6 to 19 years were different for children from 2 to 5 years. The PedsQL is applicable only to children aging 2–19. 

 The behaviour scales for different age groups were transformed using the interpolation technique where the mean of the behaviour scale scores for children from 2 to 5 years with fewer items were multiplied times the number of items for older children. This transformed mean was used in the analysis. In 18 instances, there were reports of two or three children with complex needs in the same family and only one report of parent variables. In these instances, the PMK was counted multiple times to ensure a matched number of children and parents in the analysis.

Ideally, hierarchical or multilevel modeling would be the most appropriate analytic approach because of testing within level variables (e.g., families nested within providers; children nested within families). Our data, however, are such that there is only one observation for each level. Accordingly, regression was used to examine the associations of selected child, PMK, family and treatment variables (i.e., independent variables) with the outcome child PsychQL (i.e., dependent variable), including 2-way interactions among the variables. The child, PMK, family and treatment variables for analysis were selected based on their theoretical relevance and on the contribution of the variables to the fit of the model. The fit of the model was assessed by the regression coefficient (*R* ^2^), as it measured the percentage of variation of the dependent variable explained by the model. In the final model, all possible 2-way interactions of variables were tested, and nonsignificant interactions were removed using the *Forward Stepwise Selection* technique, where the entry significant level and staying significant level were chosen to be 5% and 10%, respectively. The variables in the final model were centralized to adjust for possible multicolinearity. The normal plot was used to check the normality of the outcome (PsychQL). We examined the residuals to check possible violations of model assumptions. The interactive effect between two continuous variables was illustrated by conditional regression lines [30]. The association between one variable and the outcome was plotted as a regression line under three conditions of the other variable. The three conditions were conventionally defined as: high (any score more than 1 standard deviation above the mean), moderate (any score within 1 standard deviation to the mean) and low (any score more than 1 standard deviation below the mean). All analyses were performed using SPSS 15 (Chicago, IL).

## 4. Results

Table 1 shows the demographic characteristics of the participating families. The majority of PMK were mothers of the children (85%), born in Canada (61%) and spoke English (91%). The average PMK was 40 years, 90% were female, 85% were married/common-law, 68% were employed and the median household income was $60–$69,000. There was an even split between families residing in Simcoe (52%) and York (49%) counties. The average child age at interview was 7.94 years with 67% of the sample being male. Forty percent of the children were in preschool (up to and including Kindergarten), 35% in grades 1–5 (elementary) and 25% in grades 6 and up (junior). Sixty-one percent of children were receiving service from Community Care Access Centres and School Boards at time of entry into the CTN. The top PMK reported diagnoses for the children (Table 2) were mental and behavioural disorders (78%), diseases of the nervous system (34%), Autism (23%) and congenital malformations, deformations and chromosomal abnormalities (20%). Fifty one percent of children had more than one reported medical problem.

In Table 3, it can be seen that the study sample included children with complex needs with low physical and psychosocial quality of life as indicated by the PMK. Generally, this sample of children exhibits pro-social behaviour and low levels of anxiety, aggression and property offence behaviours. PMK positive interaction and consistency parenting practices were moderate to high in 92% and 78% of the cases, respectively. PMK hostile parenting and punitive parenting were generally low in this sample (81% and 67% scored in the low range, respectively). On average PMK report having social supports without family dysfunction and good overall life satisfaction. Forty-three percent, however, were exhibiting mild to severe symptoms of depression and anxiety (K10 > 19). For measures of processes of care, respectful and supportive care received the highest rating and providing general information received the lowest rating by PMK.

Table 4 presents the summary regression analysis. The overall variance explained by the model was 47 percent. Child's PsychQL was significantly different for different age groups. On average, junior school-aged children (grade 6 & up) had lower psychosocial scores by 4.4 points than younger children. Child hyperactivity, conduct disorder, hostile parenting and punitive parenting were statistically significantly related to PsychQL, however, these main effects had small Beta co-efficients (range −1.25 to −0.66). Comprehensive and coordinated care was statistically significantly related to the child's PsychQL as a main effect in this model. For every one point increase in CCC (0–7) there is a 2.33 point increase in child PsychQL. 

**Figure 2:**
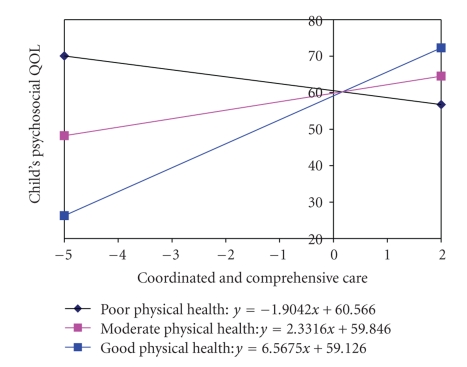
Interaction between Coordinated and Comprehensive Care and PMK Physical Health.

**Figure 3:**
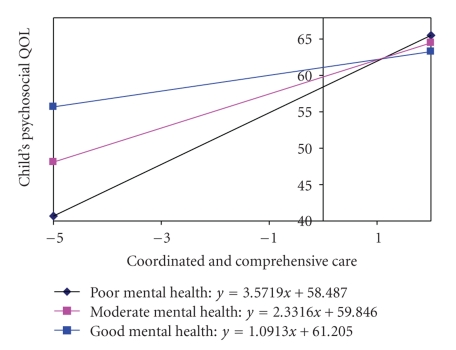
Interaction between Coordinated and Comprehensive Care and PMK Mental Health.

A number of statistically significant interactions are also presented in Table 4. Although statistically significant, many were deemed less clinically important due to low Beta co-efficients. Clinically important interactions expressed in the difference in the psychosocial quality of life scores for junior children were plotted in Figure 2 through 4. The difference in psychosocial function for younger children will show the same pattern, except that the younger children on average will have scores 4.4 points higher. The extent of CCC received was positively associated with the child's PsychQL score when the parent's physical health was moderate or good (Figure 2). In the case of parents with moderate physical health, each unit increase in the CCC score was on average associated with a 2.33% higher child PsychQL score. In the case of good physical health, each unit increase in the CCC score was on average associated with a 6.57% higher child PsychQL score. CCC, however, was negatively associated with the child's PsychQL score when the parent's physical health was poor. In these situations, each unit increase in the CCC score was associated with 1.9% lower child PsychQL score. The extent of CCC received was also positively associated with the child's PsychQL under any condition of parental mental health (Figure 3). In the case of poor PMK mental health, each unit increase in the CCC score was on average associated with a 3.57% higher child PsychQL score. In the case of moderate mental health and good mental health, each unit increase in the CCC score was on average associated with 2.33% and 1.09% higher child PsychQL scores, respectively. 

**Figure 4:**
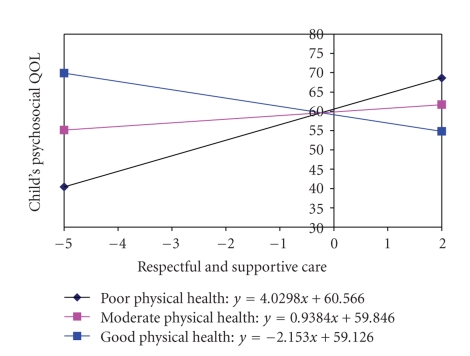
Interaction between Respectful and Supportive Care and PMK Physical Health.

The extent of RSC received was positively associated with the child's PsychQL score when the PMK physical health was moderate or poor (Figure 4). In the case of moderate PMK physical health, each unit increase in the RSC score was on average associated with a 0.94% higher child PsychQL score. In the case of poor PMK physical health, each unit increase in the RSC score was associated with a 4.03% higher child PsychQL score. RSC, however, was negatively associated with the child's PsychQL score when the parent's physical health was good and in that case each unit increase in RSC score was on average associated with a 2.2% lower child PsychQL score. 

## 5. Discussion

 This study provides original information about the psychosocial quality of life of children with complex needs and the associations and interactions of environmental influences. Quality of life scores in this sample (physical 55.77, psychosocial 50.09) were much lower than reported mean scores for healthy children (physical score 87.84, psychosocial 81.87) [3]. They were also lower than scores reported by 10 disease clusters (physical score range 64.40–85.89, psychosocial range 67.46–77.34) [3]. The scores obtained in this multidiagnoses sample were comparable to reported scores for children with Cerebral Palsy attending a CP clinic in San Diego (physical 43.19, psychosocial 55.91) [31]. Children in Varni's CP sample [31], however, were excluded if they were not able to self-report PedsQL scores. Generally the physical score in the CP group is lower likely because of the inclusion of children with quadriplegia while the psychosocial score is higher likely due to our inclusion of kids with mental and behavioural diagnoses. The low scores in this sample illustrate the multifaceted needs and issues faced by this heterogeneous group of children and youth with multiple diagnoses usually excluded from other studies. 

Children's psychosocial quality of life was influenced primarily by age in this sample. The lower psychosocial function among older children and youth could be the results of accumulating parent distress, less favorable parenting styles, child exclusion from peers and activities when in school or the possibility of discontinuities in what is measured at different ages. Also, at the time of this study rehabilitation efforts were more targeted to younger children through Early Intervention programs. There are fewer formal treatment options for older youth and perhaps older children simply decide not to pursue further efforts to improve their quality of life once they achieve greater independence. Finally, as the data is proxy reported, parent mental health expectations of their child at different ages may influence psychosocial child quality of life responses and thus overall scoring (i.e., parents of older children may have higher behavioral expectations and there are more environmental/peer influences for older children).

Child psychosocial function, as reported by parents, was also associated with negative child behaviour, reported adverse impact of the child's situation on the family, parenting practices, parent health and processes of care. Hostile and punitive parenting styles (although the prevalence in this sample was generally low) appear to be associated more with child psychosocial function than positive or consistent parenting styles. These findings are similar to previous reports in special needs populations of parent distress, negative child behaviour, and negative parenting practicing being associated with lower child PsychQL [6–9]. Also, CCC and RSC had greater association with child psychosocial quality of life than other measures of processes of care. Moore et al. [32] similarly showed that processes of care are associated with the quality of life of children with neurological conditions. These findings justify the CTN emphasis on team coordination and added psychosocial resources for parents and children.

This study also confirms that reports of simple associations between research outcomes do not give a comprehensive picture of the issues. Real life problems are rarely caused by a single underlying issue. A multitude of factors (e.g., child age, behaviour, parent mental and physical health, parenting practices, provider processes of care) and interactions among these factors are simultaneously present; therefore, care of these families requires a holistic approach that addresses all aspects of the child's environment. In addition to working with the complex child on their physical needs, some parents need help with effective parenting strategies and their own mental health in order to maximize the child's psychosocial health. Also, service providers may need to tailor their treatment approaches to different family situations. Specifically, it appears that in families receiving high comprehensive and coordinated care from service providers, the parent is in good physical health and the child's PsychQL is high. In families receiving higher RSC, however, the parent is in poorer physical health and the child's psychosocial function is high. 

On the whole, the general hypotheses of the child with complex needs being influenced by their environment (parent, family and service provider behaviours) was upheld and specified as child psychosocial quality of life varied for different environmental conditions. There appears to be a number of interactive effects at play in the environment. Further research is needed to confirm these trends and to explore the direction of the associations across real time. This data informs the child rehabilitation literature by including the role service provider's play in interaction with child and parent behaviours in explaining the psychosocial function of children with complex needs.

## 6. Limitations

Results and findings are difficult to generalize outside of this study population because other contexts may differ. The PMK in this sample were predominantly married, educated, working mothers. This study may be missing important information from working, lower educated, single parents and their children—likely those with greater need. This study probably underestimates the effects. 

Due to the cross-sectional nature of this research we do not understand the causation or directional influence of child, family and process of care variables on child's psychosocial quality of life. Longitudinal followup is needed to determine whether improvements in CCC, for example, can improve the psychosocial well-being of the child or if in fact providers need to engage actively those difficulties to reach families (i.e., families with low child PsychQL and poor parent mental health). These questions are also important in order that rehabilitation treatment efforts can be targeted and evaluated to include the needs of parents so that child quality of life can be maximized.

Sample size limitations did not allow the testing of interactions between all ecological factors and higher order interactions (3-way). Also, quality of life data were parent-reported. Generally, parents underestimate their child's quality of life compared to child self reports [3]. Therefore, the associations and interactions may vary when child self report data are used. It was not feasible to obtain self-report data from this complex needs group due to the wide range of limitations present and budget constraints of the study. Finally, clinically important change was difficult to quantify in this patient population. Research to date has not determined the minimally important difference in a diverse group of children with complex needs. 

## 7. Conclusions

Ecological factors explained 47% of the variance in child PsychQL in this complex needs child sample. Older children scored the lowest in PsychQL. It is not possible to fully understand child psychosocial quality of life in complex needs families by considering only simple associations between ecological factors. A multitude of environmental factors and interactions between these factors are simultaneously present and care of these families requires a holistic approach. Further research is needed to confirm these trends particularly in special needs children of single parents with lower education levels, generally harder to reach.

## Figures and Tables

**Table 1:** Characteristics of sample (*n* = 445).

Variable		
Respondent (PMK)		
Age (years)	Mean (SD)	40.46 (7.67)
Gender	Female, *n* (%)	400 (90)
Relationship to child	Mother, *n* (%)	380 (85)
Marital Status	Married, *n* (%)	376 (85)
Employment status	Employed, *n* (%)	302 (68)
Country of Birth	Canada, *n* (%)	338 (76)
Household language	English, *n* (%)	403 (91)
Household income	Median	$60–69,000
PMK Level of Education	Median	Completed post-secondary
PMK location of home	Simcoe, *n* (%)	230 (52)

Child		
Age (years)	mean (SD)	7.94 (4.46)
Status	Pre-school, *n* (%)	180 (40)
	Elementary, *n* (%)	154 (35)
	Junior, *n* (%)	111 (25)
Grade	Median	grade 1
Gender	Male, *n* (%)	297 (66.7)
Service Provider	Early Intervention, *n* (%)	143 (32)
	CCAC & School, *n* (%)	272 (61)
	New CTN referral, *n* (%)	30 (7)

**Table 2:** PMK reported child diagnoses (*n* = 445).

ICD-10 Diagnostic category		Count	%
A00-B99	Infectious and parasitic diseases	3	0.01
C00-D48	Neoplasm	4	0.01
D50-D89	Diseases of the blood & blood forming organs involving immune mechanism	7	0.02
E00-E90	Endocrine, nutritional and metabolic diseases	17	0.04
F00-F99	Mental and behavioral disorders	349	78.43
	Autism	104	23.37
	Unspecified Disorder of psychological development	82	18.43
	Specific developmental disorders of Speech and Language	68	15.28
	Hyperkinetic disorders (ADD/ADHD)	45	10.11
G00-G99	Disease of Nervous system	150	33.7
	Cerebral Palsy	73	16.4
	Epilepsy	38	8.54
H00-H59	Disease of eye and adnexa	20	0.04
H60-H95	Disease of the ear and mastoid process	11	0.02
I00-I99	Disease of circulatory system	11	0.02
J00-J99	Diseases of respiratory system	27	0.06
K00-K93	Disease of digestive systems	4	0.01
L00-L99	Diseases of the skin and subcutaneous tissues	3	0.01
M00-M99	Diseases of the musculoskeletal system and connective tissues	10	0.02
N00-N99	Diseases of genitourinary system	4	0.01
P00-P99	Certain conditions originating in the perinatal period	10	0.02
Q00-Q99	Congenital malformations, deformations and chromosomal abnormalities	88	19.78
	Down's syndrome	30	6.74

**Table 3:** Range, high score equivalency and mean sample scores for measured variables.

Variables	*n*	Mean (SD)	Score Range	High Score Equivalency
PedsQL (age 2+ years)				
Physical Function	429	55.77 (33.97)	0–100	Better function
Psychosocial Function	429	59.09 (18.62)	0–100	Better function

Behaviour (age 6+ years)				
Prosocial	267	10.33 (5.72)	0–20	High prosocial behaviour
Anxiety/Emotional	428	3.82 (2.97)	0–14	High emotional disorder
Indirect Aggression	425	0.96 (1.64)	0–10	High aggression
Property Offence	267	1.57 (2.01)	0–12	High offence
Hyperactivity/Inattention	425	7.50 (3.83)	0–16	High activity/inattention

Parenting				
Positive	444	15.18 (3.04)	0–20	More positive
Hostile	440	10.2 (4.93)	0–28	More hostility
Consistent	418	13.46 (3.86)	0–20	More consistency
Punitive	425	9.52 (2.05)	0–20	More punition

MPOC				
Respectful and Supportive Care	425	5.17 (1.29)	1–7	Better perception
Providing General Information	416	3.45 (1.58)	1–7	Better perception
Enabling and Partnerships	419	4.71 (1.53)	1–7	Better perception
Providing Specific Information	418	5.06 (1.43)	1–7	Better perception
Comprehensive and Cord. Care	422	4.74 (1.49)	1–7	Better perception

Social Support	429	17.58 (4.55)	0–24	More support

Impact on Family (Score transformed)	429	24.04 (9.90)	0–45	Less adverse impact

Well Being				
Family Function	429	9.22 (6.10)	0–36	High dysfunction

Parent Distress (K10)	429	20.01 (6.55)	10–50	High distress
Parent report of life satisfaction	428	1.93 (0.88)	1–5	Poor life satisfaction
Parent report of mental health	428	2.32 (1.02)	1–5	Poor mental health
Parent report of physical health	428	2.50 (1.06)	1–5	Poor physical health

**Table 4:** Regression analysis results.

Variable (range)	Coefficient	95% CI		*P*
Child Age (1 = junior, 0 = younger)	−4.40	−7.55	−1.25	**.007**
Child Hyperactivity (−7.5 to 8.5)	−1.25	−1.68	−0.83	<.0001
Child Conduct Disorder (−2 to 10)	−0.66	−1.24	−0.08	**.026**
Hostile Parenting (−10 to 18)	−0.92	−1.29	−0.54	<.0001
Punitive Parenting (−10 to 10)	−1.10	−1.84	−0.36	**.004**
Impact on Family (−24 to 21)	0.15	−0.03	0.32	.098
PMK Physical Health (−2 to 3)	0.68	−0.79	2.15	.366
PMK Mental Health (−2 to 3)	−1.34	−2.96	0.28	.106
Social Support (−17 to 7)	0.03	−0.35	0.42	.862
Coordinated and Comprehensive Care (−5 to 2)	2.33	0.62	4.04	**.008**
Respectful and Supportive Care (−5 to 2)	0.94	−1.05	2.93	.356

Child Hyperactivity × PMK Mental Health	0.42	0.06	0.77	**.021**
Hostile Parenting × Punitive Parenting	−0.16	−0.30	−0.03	**.014**
Hostile Parenting × Impact on Family	0.05	0.02	0.08	**.001**
Impact on Family × PMK Physical Health	0.15	0.02	0.28	**.029**
Impact on Family × Coordinated and Comprehensive Care	0.13	0.04	0.23	**.007**
PMK Physical Health × Coordinated and Comprehensive Care	−3.99	−5.70	−2.29	<.0001
PMK Physical Health × Respectful and Supportive Care	2.91	1.06	4.76	**.002**
PMK Mental Health × Coordinated and Comprehensive Care	1.22	0.20	2.24	**.019**

Variables were centralized by subtracting their means, so scores showed deviation from the mean.

Sample size *n* = 414 (31 cases were excluded due to partially/completely missing data).

Forward stepwise selection was used to select interaction terms: entry *P*-value = .05 and staying *P*-value = .1; *P*-value less than  .05 was considered to be statistically significant.

Goodness of fit of the model: *R*-Square = 0.4717.
